# The Effect of Particles on Electrolytically Polymerized Thin Natural MCF Rubber for Soft Sensors Installed in Artificial Skin

**DOI:** 10.3390/s17040896

**Published:** 2017-04-19

**Authors:** Kunio Shimada, Osamu Mochizuki, Yoshihiro Kubota

**Affiliations:** 1 Faculty of Symbiotic Systems Sciences, Fukushima University, 1 Kanayagawa, Fukushima 960-1296, Japan; 2 Department of Biomedical Engineering, Toyo University, 2100 Kujirai, Kawagoe, Saitama 350-8585, Japan; mochizuki@toyo.jp (O.M.); kubota548@toyo.jp (Y.K.)

**Keywords:** dielectrics, particle, NR-latex, magnetic compound fluid (MCF), sensor, electrolytic polymerization, magnetic cluster, magnetic field strength, magnetic fluid, electric conductivity, gauge factor

## Abstract

The aim of this study is to investigate the effect of particles as filler in soft rubber sensors installed in artificial skin. We examine sensors made of natural rubber (NR-latex) that include magnetic particles of Ni and Fe_3_O_4_ using magnetic compound fluid (MCF). The 1-mm thickness of the electrolytically polymerized MCF rubber makes production of comparatively thin rubber sensors feasible. We first investigate the effect of magnetic particles Ni and Fe_3_O_4_ on the curing of MCF rubber. Next, in order to adjust the electric properties of the MCF rubber, we adopt Al_2_O_3_ dielectric particles. We investigate the effect of Al_2_O_3_ particles on changes in electric current, voltage and temperature of electrolytically polymerized MCF rubber liquid, and on the electric properties under the application of normal and shear forces. By adjusting the ratio of Ni, Fe_3_O_4_, Al_2_O_3_ and water in MCF rubber with Al_2_O_3_, it is possible to change the electric properties.

## 1. Introduction

In robotics, it is sometimes necessary for sensors to be both very soft and extensible, as when they are installed in the artificial skin of robots. It is expected that robots will soon be providing humans with domestic help, in the nursery, in hospitals, etc. To enable humans to feel an affinity to robots, there has been a recent demand for a material that is similar to human skin. The rubber selected for sensors should not be vulnerable to impulsive or immense force. To create robot skin, rubber is needed that is durable so as to withstand large forces, while retaining considerable softness and expansion capability [1].

In addition, it is desirable for robot skin to have high haptic sensing like human skin. Haptic sensing can be obtained by measuring the changes in electrical resistance or dielectrics between the electrodes installed in a sensor under the application of extraneous force. The electric property changes due to the softness of the rubber material.

Therefore, it is important to examine the electric properties of soft rubber haptic sensors. The electric, as well as mechanical, property of many types of rubber sensors has already been investigated, including natural rubber (NR-latex) [2,3,4,5,6], styrene-butadiene rubber [7] and nitrile-butadiene rubber [8]. In addition, we have considered rubber sensors made of silicon oil rubber [9] and NR-latex [10,11,12] that use magnetic compound fluid (MCF), a type of magnetic filler that has electric and magnetic fields. MCF is a colloidal fluid containing magnetic particles of Ni and Fe_3_O_4_ [13]. It is useful for application in polymers to improve the sensor in a number of ways. MCF was devised by Shimada in 2001 [13] as an intelligent fluid containing Fe_3_O_4_ 10-nm order thick sphere particles coated with oleic acid, as well as 1-μm order thick metal particles such as Ni, etc. Fe_3_O_4_ particles are dispersed in magnetic fluid (MF) with a mixing solvent such as water, etc., which is responsive to a magnetic field. When a magnetic field is applied, owing to the bonding role among the metal and Fe_3_O_4_ particles, numerous magnetic clusters form [14]. These magnetic clusters make MCF useful for engineering applications, including in polishing, damper, and composite material.

Rubber that includes MCF as a composite material is called MCF rubber. The magnetic clusters in MCF rubber produce high electric conductivity at a very small pressure. The mechanism of electric conductivity has been explained mainly by the percolation [15] and tunnel theories [16], etc. In the percolation theory, the percolation threshold is a very significant factor. However, when the aspect ratio of particle shape becomes large due to the aggregation of particles, the percolation theory is not applicable, because the threshold becomes small in spite of enhanced electric conductivity. In the tunnel theory, when voltage is applied to the material, the phenomenon of electrons jumping through a non-conductive material barrier can be explained by the tunnel effect. In the case of MCF rubber made of silicon oil rubber, the magnetic clusters exhibit extreme aggregation [9]. Therefore, the tunnel theory is thought to be more suitable. In contrast, for MCF rubber made of NR-latex, the ions inside the MCF rubber become closer, in addition to exhibiting the tunnel effect, resulting in electric conductivity changes. Thus, the existence of magnetic clusters in MCF rubber creates a filler with electrical conductivity.

In the case of MCF rubber made of NR-latex, the application of an electric field works to electrolytically polymerize the MCF rubber sufficiently to create high electric conductivity, as shown in our previous studies [10,11,12]. In general, human skin is well-known to have a haptic sensitivity of 1.96 × 10^4^ Pa, a characteristic value found in number of studies [17]. In contrast, electrolytically polymerized MCF rubber has a haptic sensitivity of 2 × 10 Pa, corresponding to the measured value of extraneous pressure at the moment that its electric property begins to change. The magnetic clusters induce this high haptic sensitivity as follows: when an electric field is applied to MCF rubber liquid, solid MCF rubber is created on an anode as a thin film. When a magnetic field is added to the anode, MCF rubber grows thicker along the application line of the magnetic field. As a result, as the alignment of C=C bonds of NR-latex is promoted by the alignment of magnetic clusters, the electric conductivity of the MCF rubber is enhanced. Incidentally, the electric conductivity of MCF rubber made of NR-latex is higher than that made of silicon oil rubber [9]. In the case of NR-latex type MCF rubber, the electric conductivity resulting from electrolytic polymerization is added to the tunnel and ion effects described above. Therefore, in the present study, we deal with NR-latex type MCF rubber.

The aim of the present study is to develop a soft rubber made of NR-latex for application as a high-haptic sensor in artificial skin. It is important to investigate how to control sensitivity in the production process. The Ni and Fe_3_O_4_ particles involved in filler MCF rubber are a significant factor. Here, we dealt with 1 mm thick electrolytically polymerized NR-latex MCF rubber since it is more feasible for the production of a sensor [10,11,12]. It was also important to investigate the effect of particles on the curing of MCF rubber. In our previous studies [10,11,12], we investigated the effects of mass concentration and applied magnetic field strength on electric and mechanical properties. The effect of particles on curing, however, have not yet been clarified. Therefore, we first examine the effect of Ni and Fe_3_O_4_ magnetic particles on the curing of 1 mm thick electrolytically polymerized NR-latex MCF rubber.

As is well known, in terms of dielectrics, sensor characteristics can be divided into four types: paraelectrics, piezoelectrics, pyroelectrics, and ferroelectrics. Therefore, if dielectric materials are often used as particles in polymer blends (corresponding to filler), the sensor could have a range of possible sensitivities. Moreover, the use of dielectric materials as filler is one of the best ways to enhance the electrical, thermal, mechanical or curing properties of a composite material [18,19,20,21,22].

However, more study is needed on the available properties of MCF rubber mixed with dielectric particles for sensors. In this study, we therefore investigate ways of controlling the electric properties of MCF rubber. We adopt the dielectric particle Al_2_O_3_ as filler, and decrease electric conductivity by varying the mixture of dielectric materials. We also examine the effect of the Al_2_O_3_ particle on electric conductivity, using the same measurement apparatus used in our previous studies [10,11,12].

## 2. The Effect of Curing on Particles

The New Method used to produce MCF rubber has been described previously [10,11,12]. Briefly, MCF rubber liquid was poured between two metal plates (stainless steel, 35 mm square, 1 mm thick), and permanent magnets were applied to the inside of plates. Magnets were 15 mm × 10 mm in size, and 5 mm thick. The magnetic field strength was varied at 188, 312, 490, and 721 mT. A constant electric field was also supplied between the plates at 6 V and 2.7 A, at 30 min periods. The experiments were conducted under atmospheric room conditions. The plates were held apart using a 1 mm spacer as an electrodes gap. When the electric field was applied, the MCF rubber liquid was vulcanized.

We used five types of MCF rubber (Types 1–5) with different mass concentration ratios of magnetic particles Ni and Fe_3_O_4_, as shown in Table 1. Here, Ni was a powder with μ-m order thick twig-shaped particles (No. 123, Yamaishi Co., Ltd., Noda, Japan), MF was 50 wt% Fe_3_O_4_ (M–300, Sigma Hi–Chemical Co., Ltd., Tsutsujigasaki, Japan), and NR-latex was from Rejitex Co. Ltd. (Atsugi, Japan). Type 1 corresponds to the MCF rubber liquid used in our previous reports [10,11,12].

Table 2 presents photographs of the spikes in Types 1–5 MCF rubbers due to the application of the magnetic field only. Because the numerous aggregated particles were separated into solid and liquid states, with a larger magnetic field, MCF rubber liquid presented distinctly solid and liquid states. When the magnetic field was smaller, the density of the magnetic clusters was reduced, and the MCF rubber liquid presented mixed solid-liquid states. The larger the mass concentration of MCF rubber, the more solid the state of the MCF liquid. The phenomenon shown in Table 2 is related to the enhancement of viscosity by application of the magnetic field. In general, MCF viscosity is increased together with magnetic field strength, and the mass concentration of MCF, and behaves as a non-Newtonian fluid due to the magnetic clusters of aggregated particles, as shown previously [13]. It was considered that the present results were obtained by the same aggregation.

**Figure 1:**
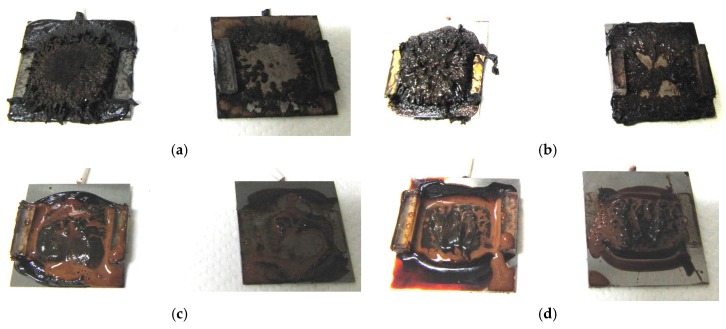
Photographs of Type 2 vulcanized MCF rubber after electrolytic polymerization by four different magnetic field strengths; the left column shows the anode surface, and the right column the cathode surface at: (**a**) 188 mT, Solidification 1; (**b**) 312 mT, Solidification 2; (**c**) 490 mT, Solidification 3; and (**d**) 721 mT, Solidification 4.

Figure 1 shows electrolytically polymerized MCF rubber on the anode (left column) and cathode (right column) after application of the electric field to Type 2 MCF rubber. Vulcanization can be classified into 4 types: all areas of the rubber are vulcanized to dryness (Figure 1a; Solidification 1); all areas of the rubber are vulcanized, but remain wet (Figure 1b, Solidification 2); about half of the rubber is vulcanized and wet (Figure 1c; Solidification 3); and only a part of the rubber is vulcanized and the rest remains liquid (Figure 1d; Solidification 4). At smaller magnetic field strength, MCF rubber is vulcanized more rigidly, like a dried solid. Table 3 compares vulcanization after electrolytic polymerization by four different magnetic field strengths in all five rubber types, including Type 2. As may be seen by the distribution of solidification types, the MCF rubber at larger magnetic field strengths did not solidify easily, remaining liquid at all mass concentrations. With a smaller magnetic field strength, on the other hand, both small and large mass concentrations of MCF rubber were vulcanized more rigidly. This was due to the density of the magnetic clusters.

Figure 1 and Table 3 show that when magnetic field strength was large, MCF rubber liquid remained in a liquid state before electrolytic polymerization, and was not sufficiently vulcanized by electrolytic polymerization. This tendency did not depend on the mass concentration.

On the other hand, with a small magnetic field strength, the MCF rubber liquid gradually became solid before electrolytic polymerization, and was more vulcanized by polymerization. In addition, the smallest and largest mass concentrations were more solidified by electrolytic polymerization. Thus, results indicated that when MCF rubber liquid had mixed solid and liquid states, even if it presented as extremely solid or liquid, it was more vulcanized by electrolytic polymerization.

## 3. The Effect of Dielectric Particle

Our previous investigations [10,11,12] demonstrated that the 1 mm thick MCF rubber produced by the new electrolytic polymerization method has high electric conductivity, on the order of 10^−4^ Ω·m. In comparison, the leading commercial NR-latex pressure-sensitive electrically conductive rubbers (PSECR) has 1-ordered Ω·m after thermal degradation. Metals such as silver and copper have 10^−7^-ordered Ω·m. In the case of plastic-type electric conductive polymers, polyphenylene-based conductive plastic doped with ASF_5_ has 10^−2^ ordered Ω·m. MCF rubber can therefore have electric conductivity closer to that of metal, depending on the dopant and filler. However, robotics occasionally requires sensors that are able to respond to sensitivity over a range of electric conductivity; thus, adjustable electric conductivity is needed. As mentioned, higher electric conductivity has been well-developed, while degradation of electric conductivity requires further investigation. In order to decrease electric conductivity, it is best to use dielectric material. 

We used 3 μm sphere particles of Al_2_O_3_ (CR, Struers Co., Ltd., Tokyo, Japan) in the three ingredient combinations listed in Table 4. Here, MF was 40 wt % Fe_3_O_4_ (W-40, Taiho Kozai Co. Ltd., Tokyo, Japan). It was separately confirmed that the effect of different kinds of MF (like W-40 and M-300) on the electric properties of electrolytically polymerized MCF rubber was not an issue.

**Figure 2:**
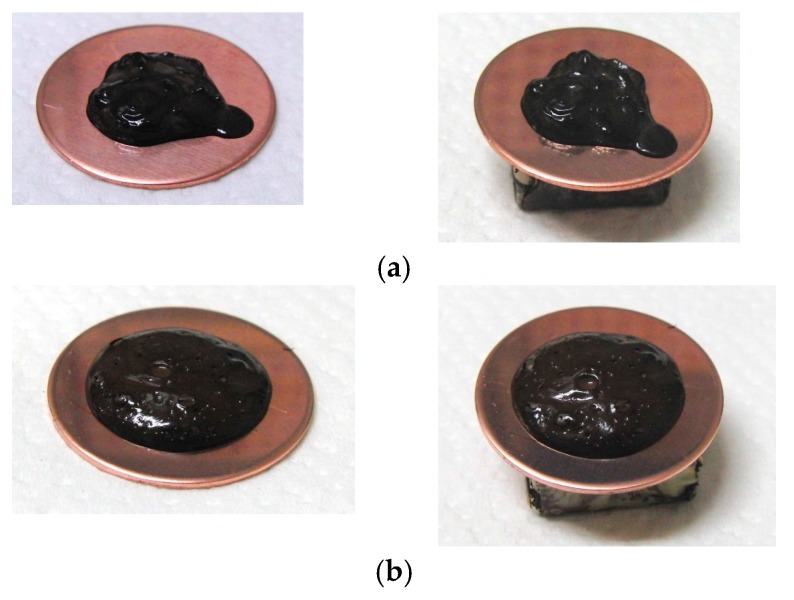

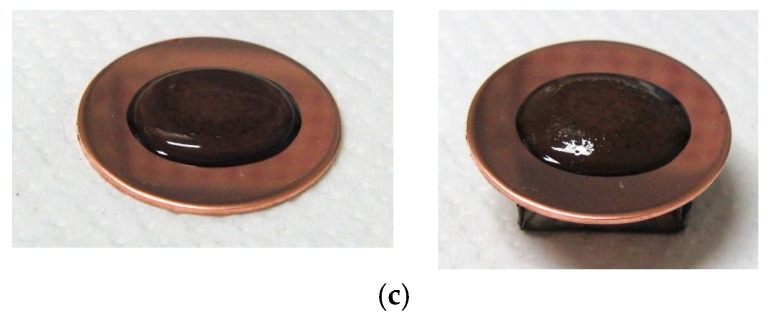
Photographs of MCF rubber Types 6–8 with Al_2_O_3_ dielectric particles before electrolytic polymerization, without the application of a magnetic field (left column) and with application (right column): (**a**) Type 6; (**b**) Type 7; (**c**) Type 8.

Figure 2 presents photographs of MCF rubber liquids of Types 6–8 before electrolytic polymerization, both without (left column) and with (right column) the application of a magnetic field of 188 mT. Unlike Type 1 in Table 2, with the Al_2_O_3_ particles, MCF rubber liquid close to a solid state did not change to a mixed solid/liquid state either with or without the magnetic field. The Al_2_O_3_ particles kept the MCF rubber liquid closer to a solid state.

**Figure 3:**
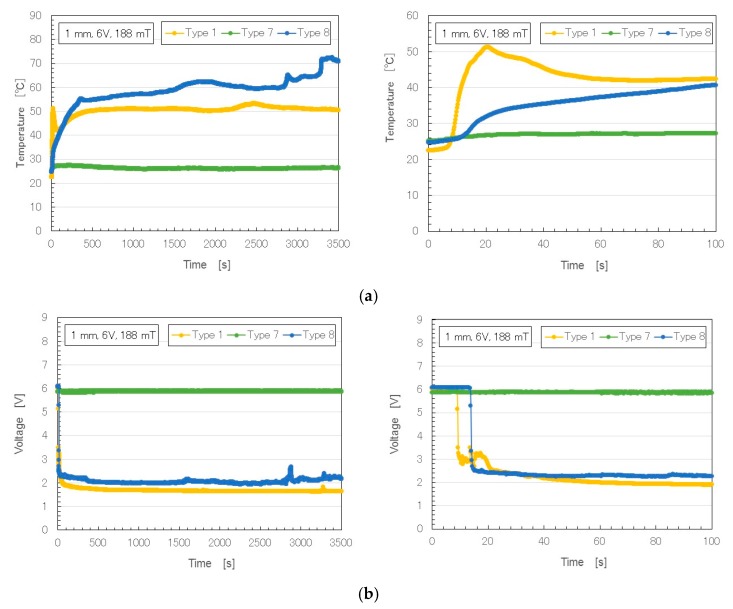

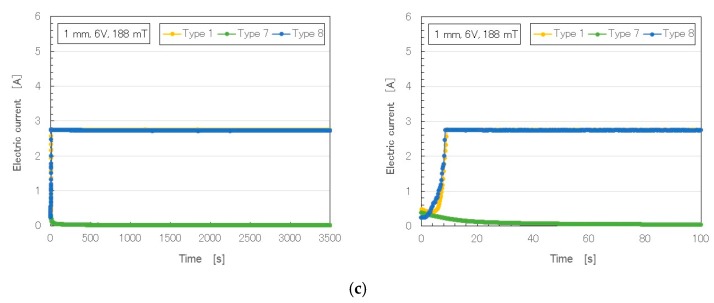
Time changes of temperature, voltage and electric current of MCF rubber under electrolytic polymerization: (**a**) temperature; (**b**) voltage; (**c**) electric current.

We measured the voltage and electric current flowing inside the MCF rubber liquid under the application of both electric and magnetic fields, as described in our previous studies [10,11,12]. Figure 3 shows the changes over time in temperature, voltage and electric current (6 V, 2.7 A, and 188 mT) in rubber with Al_2_O_3_ compared to that without (Type 1). Compared to Types 6–8, Type 1 rubber was the same weight of NR-latex, MF and Ni. In our previous study of Type–1 MCF rubber liquid [10,11,12], electricity was first applied at a voltage held constant at 6 V, and the electric current was zero, because the MCF rubber had not yet vulcanized; the electric resistance of the liquid MCF rubber was large. As the MCF rubber began to be polymerized, both electrolytically and thermally, the electric current and temperature increased. At the end of electrolytic polymerization, the electric current and temperature reached a constant, and voltage changes decreased to a constant. From the constant values of the electric current and voltage, the electric resistivity of the electrolytically polymerized MCF rubber could be estimated.

In contrast, in the MCF rubber with Al_2_O_3_, the temperature and electric current did not change as a result of the addition of the dielectric material. Here, a comparison of Types 7 and 8 shows that both temperature and electric current increased when water was added. Water plays a significant role in electrolytic polymerization. However, Types 7 and 8 MCF rubbers were vulcanized dry. The aridity of Type 8 MCF rubber was distinctly greater than that of Type 7. Electrolytic polymerization also vulcanized all of MCF rubber Types 6–8, according to the solidification phenomena categories in Table 3: Type 6 was Solidification 1; Type 7, Solidification 1 intermediate between Types 6 and 8; and Type 8, Solidification 1 with several cracks. When Al_2_O_3_ was included in electrolytically polymerized MCF rubber, it became like a rigid solid.

We investigated the electric properties of vulcanized MCF rubber under the application of pressure and shear force. We used the same experimental apparatus described in our previous study [10,11,12]. When we assume realistic robot movement, both the force applied transversely to the robot’s artificial skin (relevant to the haptic sensing of touch), and the shear force applied parallel to the artificial skin (related to the sensation of scraping or caressing), are significant. The former corresponds to our previous normal force experiments (NFE), and the latter to previous shear force experiments (SFE) [10,11,12].

First, we measured the electric current, voltage, or electrical resistance between the opposing electrodes by applying a normal force to the vulcanized MCF rubber. By accomplishing the data, an electric power 10 V was supplied and 1.8 kΩ electric resistance used. The MCF rubber placed between the two 7 mm stainless steel square electrodes was vulcanized. The upper electrode was moved to touch the lower one by an actuator. The pressing speed was 10 mm/min. MCF rubber test specimens were 1 mm thick, compressed between the counter electrodes. For convenience, this experimental procedure is called by NFE.

Second, in order to measure the electric current, voltage, or electrical resistance within the vulcanized MCF rubber under a shear force, we used another experimental apparatus. As with the NFE, the power 10 V was supplied and the electric resistance of 1.8 kΩ used. Between the two electrodes of the stainless plate, both tips of the vulcanized MCF rubber were placed. The MCF rubber touched a rubbing flat plate. It had a *R_a_* with 20.86 μm, *R_y_* with 199.9 μm, and *R_q_* with 26.89 μm surface roughness, and was moved parallel to the material surface by an actuator. The speed was 5 mm/s, and a sweeping distance was 50 mm. Between the MCF rubber and the acrylic resin body, a hard, non-electric body with 0.5 mm diameter was interposed, and then the rubber could be contacted exactly. This touching method was found to be more effective in a sensor, as shown in the previous study [10,11,12]. Because the contact area was smaller than the MCF rubber as a whole, and with a slight bend, the sensing then increases. The MCF rubber test specimens were 1 mm thick. For convenience, this experimental procedure is called by shear force experimental (SFE).

**Figure 4:**
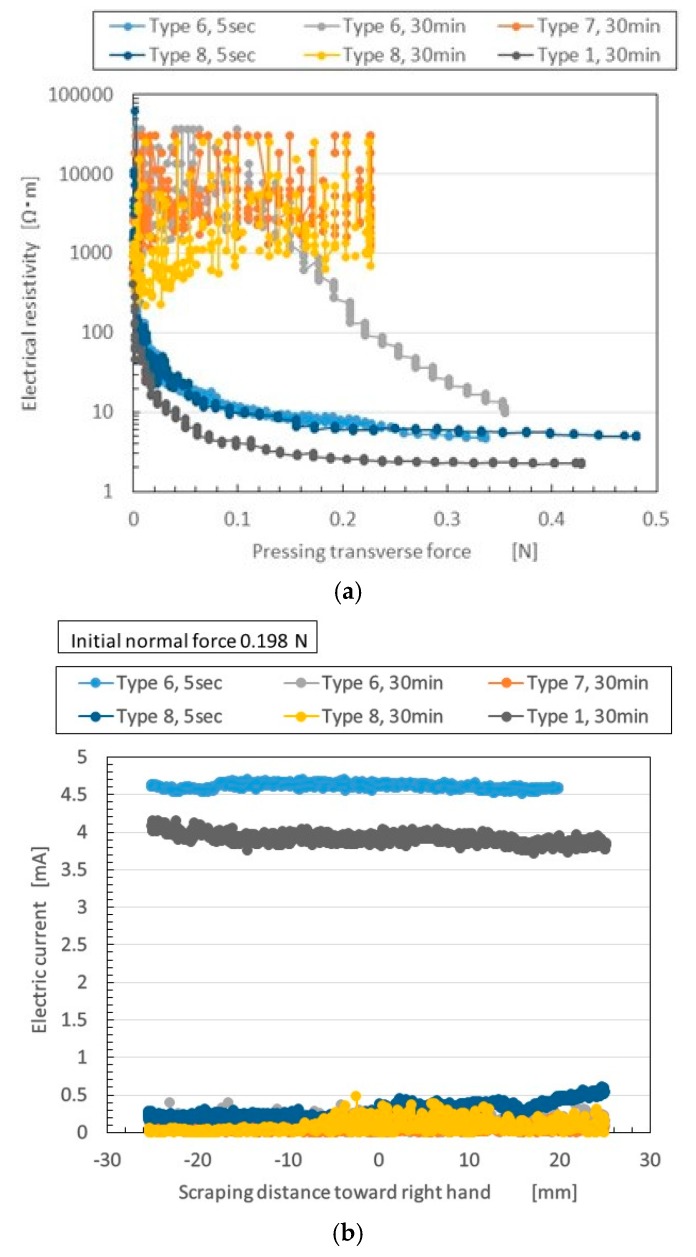
Electric characteristics of MCF rubber with Al_2_O_3_: (**a**) electric property in normal force experiments (NFE); (**b**) that in shear force experiments (SFE).

Figure 4 compares the electric properties of Types 6–8 MCF rubber to those of Type 1. The figure also plots electric property by elapsed time of vulcanization at electrolytic polymerization: 5 s vs. 30 min.

In the case of the NFE in Figure 4a, changes in electrical resistivity appear when the normal force is increasing. The fluctuation of electrical resistivity varies according to the ratio of Al_2_O_3_ and water. In Types 7 and 8 with 30 min (and Type 6 with 30 min at less than 0.1 N), the fluctuation of electrical resistivity is large and does not decrease much with increasing normal force. Aside from these types, electrical resistivity decreased. The characteristics were sufficient to create a switching effect in a sensor—when normal force reaches some targeted value, the electric current can start flowing.

When the MCF rubber included Al_2_O_3_ in a range of less than 0.15 N, electrical resistivity worsened with longer vulcanization time. However, Type 1 without Al_2_O_3_ did not deteriorate. This was thought due to the hard aridity of the Al_2_O_3_ MCF rubber by electrolytic polymerization. Because Al_2_O_3_ is hygroscopic, the water in the MCF rubber is not lost in shorter electrolytic polymerization; however, it is lost with lapsed electrolytic polymerization. The electric conductivity depends on the ions generated by the water or molecular structure of the NR-latex. In addition, when water is involved in the MCF rubber, rubber temperature under electrolytic polymerization increases, as shown in Type 8 in Figure 2, and it becomes extremely dry with aggravated electric conductivity. When the ratio of Ni to Al_2_O_3_ was increased, however, as in Type 6, electrical resistivity to pressure changes decreased, even at pressures of more than 0.15 N. Thus, electrolytic polymerization can be altered by adjusting the ratio of Ni and Al_2_O_3_ in the MCF rubber.

Next, we investigated the shorter vulcanization time of 5 s. Al_2_O_3_ degrades electric conductivity, whereas water improves it. The deterioration changes the ratio of water, Ni, and Al_2_O_3_.

In addition, the characteristics shown in Figure 4a are relevant to an effective metric of sensing, the gauge factor (GF). The GF denotes how changes in electric resistance depend on strain. GF is evaluated with strain as follows:

GF = (Δ*R*/*R_o_*)/ε (1)

where Δ*R* is the change in electric resistance with strain, *R_o_* is the initial value of electric resistance, and ε is the strain. Strain has been investigated in polymers [23,24,25] and rubber [26,27,28,29,30] used as soft sensors. The NR-latex has been utilized [27,28,29,30]. Many of the investigations of GF have focused on the strain, and there have been few investigations of GF in connection with compression. However, the GF at compression is also important for sensors. When thickness is less than perpendicular length, the GF tends less to compression and more to extension, because the sensor is frequently used as a thin film. Here, we investigate the GF of the MCF rubber at compression.

By expanding Equation (1) to the experimental condition of compression, GF can be considered as the gradient of the curve of Figure 4a. This is because Equation (1) is the tangential value of the curve of the relation between electrical resistivity and pressing force at some pressure when the electrical resistivity is a function of the pressing force. As the pressure was enhanced, the tangential value could be approximated to the gradient of the curve of Figure 4a. Rubber Types 6 and 8 with 5 s, and Type 1 with 30 min, showed substantial changes in GF under enhanced pressure. This was due to the switching effect, as explained by the tunnel effect, and to the ions inside the MCF rubber getting closer, resulting in electric conductivity changes.

**Figure A1:**
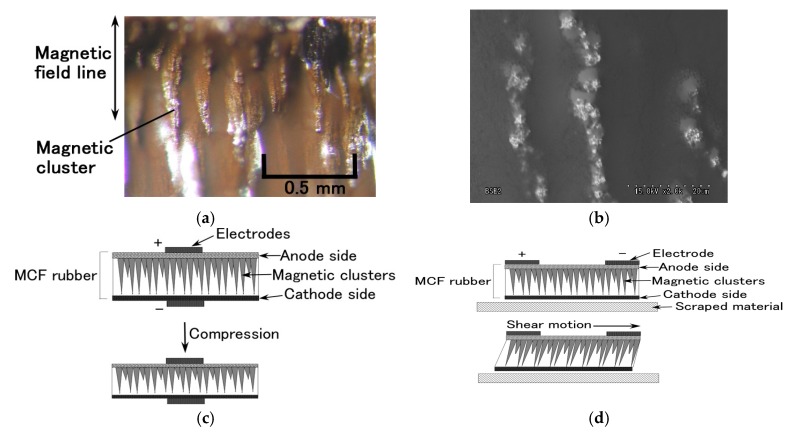
Cross section of electrolytically polymerized MCF rubber: (**a**) microscopic image of magnetic clusters [31]; (**b**) image magnifying a part of needle-like magnetic cluster in (**a**) by SEM, ×2000; (**c**) schematic diagram of MCF rubber transformed by compression [31]; (**d**) schematic diagram of transformed MCF rubber by shear force.

The electrolytically polymerized MCF rubber has many needle-shaped magnetic clusters, like those shown in Figure A1a,b, whether or not Al_2_O_3_ particles are involved. The MCF rubber was then transformed by pressing, as shown in Figure A1c. As the electric current flowed easily along the aligned magnetic clusters and the magnetic clusters got closer, the electric current increased exponentially according to the enhanced pressure. The oleic acid coating the Fe_3_O_4_ and Al_2_O_3_ particles are non-conductive. Due to their optimum mass concentration, the electric current cannot flow easily, and electric resistance is large to the pressure. However, the electrons jump between the non-conductive materials. Electric resistance changes decrease exponentially by pressing, and the phenomena for compression can be explained by tunnel theory, just like for strain [24,26].

Meanwhile, in the NFE in Figure 4b, the abscissa of the figure represents the distance the MCF rubber was scrapped from left to right. The greater the magnitude and range of changes in the electric current, the more sensitive the sensor. Therefore, we paid attention to the quantitative value of the electric current. Different results were found for SFE in Figure 4b. Type 6 with 5 min had larger electric current than any other type, similar to Type 1. This suggests that for MCF rubber involving water, the most significant factors are the ratio of Ni to Al_2_O_3_, and vulcanization time. This is not due to the independent existence of Al_2_O_3_ and water, but rather to the difference in the morphological structure of the MCF rubber by electrolytic polymerization to the normal and shear deformation. In the SFE, the magnetic field was applied along the direction of the electrolytic polymerization between the electrodes, which was traversed to the deformation by the shear motion. In contrast, in the NFE, it was in the same direction as the deformation by compression. The morphological structure of the MCF rubber by electrolytic polymerization can be proposed as three layers, and the magnetic clusters of Ni and Fe_3_O_4_ and NR-latex combined into complicated structures, as shown in previous studies [10,11,12]. Especially in the middle layer of the MCF rubber, between the anode- and cathode-side surfaces, there are many needle-like magnetic clusters (Figure A1a). These clusters are transformed by shear motion, as shown by Figure A1d. Although the electric current can flow along the magnetic clusters, it can flow more easily between very close magnetic clusters, by the tunnel effect (Figure 4a). Therefore, the movement and path of electrons or ions through MCF rubber is different on normal- and shear-deformed MCF rubber.

## 4. Conclusions

We first clarified the effect of MCF, Ni and Fe_3_O_4_ magnetic particles on the curing of electrolytic polymerization of the MCF rubber. We found that MCF rubber maintains a more liquid state before electrolytic polymerization at a larger magnetic field strength, and is not vulcanized sufficiently by electrolytic polymerization. This tendency does not depend on the mass concentration. In contrast, at a smaller magnetic field strength, MCF rubber liquid gradually assumes a more solid state before electrolytic polymerization, and then is more vulcanized by polymerization. In addition, at the smallest or largest mass concentrations, it is more solidified by electrolytic polymerization. These results indicate that when MCF rubber liquid has mixed states of solid and liquid, even if it presents strongly one way or the other, it will be more vulcanized by electrolytic polymerization. The reason for these results is related to the density of the magnetic clusters, which is determined by the magnetic field strength and the mass concentration of magnetic particles.

Next, in order to adjust electric conductivity, we determined the best way to use the dielectric material, Al_2_O_3_. Temperature and electric current under electrolytic polymerization do not change by the addition of Al_2_O_3_. However, MCF rubber with Al_2_O_3_ is electrolytically polymerized, like a rigid solid. In contrast, by involving water, the temperature and electric current under electrolytic polymerization increase. Water plays a significant role in electrolytic polymerization. The MCF rubber can become so dry that its electric conductivity to pressure is aggravated. However, the ratio of Ni to Al_2_O_3_ in MCF rubber is increased, while the electrical resistivity to pressure changes decreases. Thus, electrolytic polymerization changes by adjustments to the ratio of Ni, Al_2_O_3_, and water in the MCF rubber.

Regarding electric property, for MCF rubber with Al_2_O_3_, electrical resistivity to pressure worsens with longer vulcanization time. With shorter vulcanization time, due to more water, electric conductivity to pressure improves. The deterioration changes the ratio of water, Ni, and Al_2_O_3_. On the other hand, the electric property under shear motion is different from that under pressure. Electric sensitivity under shear motion is enhanced by a larger ratio of Ni to Al_2_O_3_, and a shorter vulcanization time.

As a result, MCF rubber with Ni and Fe_3_O_4_ magnetic particles only does not exceed the solidification achieved by MCF rubber that includes Al_2_O_3_. However, by adjusting the ratio of Ni, Fe_3_O_4_, Al_2_O_3_, and water, electric properties of MCF rubber with dielectric particle Al_2_O_3_ to pressure and under shear motion can be changed. This technique of adjusting the electric properties is significant for the requirement of sensing at an overall force range in robotics.

## Figures and Tables

**Table 1:** Mass concentration of magnetic compound fluid (MCF) rubber liquid used in this study and ratios of mass concentration of magnetic particles and natural rubber (NR)-latex.

	Type 1	Type 2	Type 3	Type 4	Type 5
**NR-Latex (g)**	6	6	6	6	6
**MF (g)**	1.5	2	3	3	3
**Ni (g)**	6	9	11	16	21
**Mass Concentration of Magnetic Particles of Ni and Fe_3_O_4_ (wt %)**	48.8	58.8	62.5	70.0	75.0

**Table 2:** Photographs of spikes in Type 1–5 MCF rubbers before electrolytic polymerization by application of four different magnetic field strengths.

	Type 1	Type 2	Type 3	Type 4	Type 5
188 mT	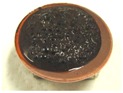	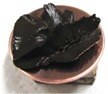	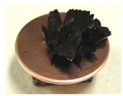	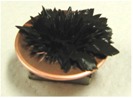	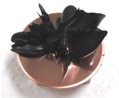
312 mT	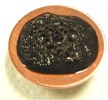	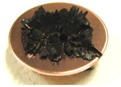	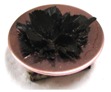	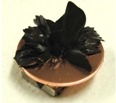	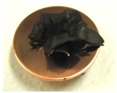
490 mT	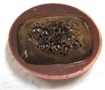	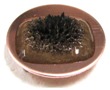	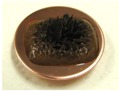	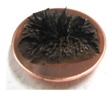	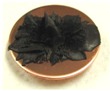
721 mT	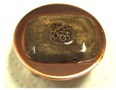	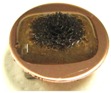	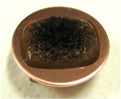	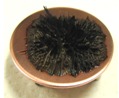	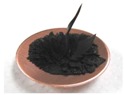

**Table 3:** Phenomena of Types 1–5 vulcanized MCF rubbers after electrolytic polymerization at four different magnetic field strengths.

Magnetic Feld (mT)	Type 1	Type 2	Type 3	Type 4	Type 5
188	Solidification 1	Solidification 1	Solidification 2	Solidification 2	Solidification 1
312	Solidification 1	Solidification 2	Solidification 3	Solidification 3	Solidification 1
490	Solidification 3	Solidification 3	Solidification 4	Solidification 4	Solidification 3
712	Solidification 4	Solidification 4	Solidification 4	Solidification 4	Solidification 4

**Table 4:** Ingredient combinations of MCF rubber liquids Types 6–8 with dielectric particle.

	Type 6	Type 7	Type 8
**Ingredient**	NR-latex: 6 g	NR-latex: 6 g	NR-latex: 6 g
	MF: 1.5 g	MF: 1.5 g	MF: 1.5 g
	Ni: 6 g	Ni: 2 g	Ni: 2 g
	Al_2_O_3_: 3 g	Al_2_O_3_:4 g	Al_2_O_3_: 4 g
	Water: 2 g		Water: 2.8 g

## Appendix A

Figure A1a [31] and A1b show images at cross-sections of electrolytically polymerized MCF rubber by microscope and scanning electron microscope (SEM), respectively. The MCF rubber consists of 12-g Ni, 3-g MF (M-300) and 12-g NR-latex. The perpendicular direction is the applied magnetic field line, which was the same as the electric field. The magnetic clusters form along the magnetic field line. The needle-like magnetic cluster consists of many chains by the aggregation of Ni and Fe_3_O_4_ particles, as shown by Figure A1b. For the NFE, the compressed MCF rubber is delineated by schematic diagram as shown by Figure A1c [31], and for the SFE, the transformed MCF rubber was produced by the application of shear force as shown in Figure A1d.
